# *In-vitro* antibiotic resistance phenotypes of respiratory and enteric bacterial isolates from weaned dairy heifers in California

**DOI:** 10.1371/journal.pone.0260292

**Published:** 2021-11-24

**Authors:** Sarah Depenbrock, Sharif Aly, John Wenz, Deniece Williams, Wagdy ElAshmawy, Kristin Clothier, Heather Fritz, Gary McArthur, Meera Heller, Munashe Chigerwe

**Affiliations:** 1 Department of Veterinary Medicine and Epidemiology, University of California, School of Veterinary Medicine, Davis, California, United States of America; 2 Veterinary Medicine Teaching and Research Center, School of Veterinary Medicine, University of California Davis, Tulare, California, United States of America; 3 Department of Population Health and Reproduction, School of Veterinary Medicine, University of California, Davis, California, United States of America; 4 Field Disease Investigation Unit, College of Veterinary Medicine, Washington State University, Pullman, Washington, United States of America; 5 Department of Internal Medicine and Infectious Diseases, Faculty of Veterinary Medicine, Cairo University, Giza, Egypt; 6 California Animal Health and Food Safety Laboratory, University of California, School of Veterinary Medicine, Davis, California, United States of America; 7 Owner, “Swinging Udders Veterinarian Services”, Galt, California, United States of America; Cornell University, UNITED STATES

## Abstract

Antimicrobial drug (AMD) use for bovine respiratory disease (BRD) continues to be concerning for development of antimicrobial resistance (AMR) in respiratory and enteric bacteria of cattle. This study aimed to provide data regarding AMR in respiratory isolates, and identify relationships between respiratory and enteric AMD susceptibility, in weaned dairy heifers. A cross-sectional study was performed between June of 2019 and February 2020, on 6 calf rearing facilities in California. Deep nasopharyngeal and rectal swabs were collected from 341 weaned heifers and submitted for selective bacterial culture and AMR testing. *Mannheimia haemolytica*, *Pasteurella multocida*, and *Histophilus somni* were selectively isolated from respiratory samples; *Escherichia coli* and *Enterococcus* spp. were selectively isolated from rectal swabs. Minimum inhibitory concentrations (MIC) were determined for selected isolates against 19 AMD. The proportion of resistant isolates was calculated using Clinical Laboratory Standards Institute (respiratory) or USDA NARMS (enteric) breakpoints; when no applicable breakpoint was available, the distribution of MIC was described and compared. Association between AMR in a calf’s respiratory isolate and a higher or lower MIC of the matched enteric isolates was determined. More than 50% of *P*. *multocida* isolates were resistant to each of 7 AMD commonly used to treat BRD (florfenicol, gamithromycin, tildipirosin, tilmicosin, danofloxacin, enrofloxacin and tetracycline). Resistance in respiratory isolates was only associated with higher matched enteric MIC for gamithromycin and tulathromycin. Multidrug resistance was reported in >70% of *P*. *multocida* and *M*. *haemolytica* isolates. Antimicrobial resistance, including multidrug resistance, in respiratory isolates appears to be widespread in weaned dairy heifers; this finding has not previously been reported and raises concern for the future efficacy of AMD used to treat respiratory diseases in weaned dairy heifers. Enteric bacterial MIC appear to have limited direct association with respiratory isolate AMR classification.

## Introduction

Bovine respiratory disease (BRD) is a significant health problem, with serious welfare and economic implications in the US cattle industry. Morbidity due to BRD in weaned dairy heifers in the US was most recently estimated at 11% and has a negative economic impact on production [1–4]. Short term economic losses in dairy calves associated with respiratory disease are estimated at $42.15 per calf affected [1]. The BRD complex is a multifactorial disease that depends on the interaction among host immunity, environmental factors such as sanitation and heat stress and pathogen factors such as virulence [5–9]. Despite preventive methods, including vaccination against respiratory pathogens, and treatment or metaphylaxis using antimicrobial drugs (AMD), treatment failures are frequent, and BRD remains highly prevalent among herds [1, 2, 10–12]. One pathogen factor that might contribute to treatment failure is the increasing trend of antimicrobial resistance (AMR) among BRD pathogens [13, 14]. In addition to negatively affecting treatment success for BRD, AMR in livestock also presents a health concern for livestock workers [15–17], consumers [18, 19] and the environment [20–22].

Heifer rearing represents a significant portion of California’s $7.3 billion dairy industry, but the scope and impact of AMR in California’s weaned dairy heifer population has not previously been described [2, 23]. This animal population consists of heifers weaned off milk or milk replacer and then moved to comingled (group) pens where they are fed a solid feed diet. The majority of weaned heifer deaths are due to respiratory disease [12]. Weaned heifers represent a valuable opportunity to study calf-hood BRD and AMR after prior hutch treatments, comingling, and any recent BRD treatment in the weaned pens. Both respiratory and enteric bacterial populations are of interest in the study because respiratory and enteric bacteria have been reported to share genetic mechanisms of AMR [24]. Dispersion of AMR may also occur in the environment; for example, deposition of manure from dairies on soils has been demonstrated to alter AMR genes in soil [21, 22]. Additionally, there may be a zoonotic risk from resistant enteric organisms to farm workers [25]. There is concern that these resistant populations may persist in the calves’ gastrointestinal tract, however the duration of AMR in enteric organisms following AMD therapy in cattle appears to change over time, and varies depending on the drug and population investigated [26, 27].

The aims of this cross-sectional study were to describe the proportion (to provide an estimate of prevalence) of respiratory isolates classified as AMR, describe the Minimum Inhibitory Concentration (MIC) distribution of enteric isolates from the same animals, describe the relationship between AMR in BRD bacterial isolates and higher or lower MIC of enteric marker bacteria using prevalence odds ratios, and define which multi-drug resistance (MDR) patterns are most prevalent in respiratory samples from weaned dairy heifers in California.

The hypotheses of this study are: 1) AMR is detectable in respiratory isolates across all study farms to all AMD classes with applicable breakpoints for defining AMR; 2) the most prevalent bacterial AMR phenotypes in respiratory isolates is resistance to tetracycline and macrolide class AMD based on historic anecdotal reports of frequent use of these drugs in the study population, and; 3) that the presence of specific AMR in respiratory pathogens is associated with higher MIC to similar AMD in enteric isolates from the same animals.

## Materials and methods

### Study design and study herds

A cross-sectional study was carried out between June of 2019 and February 2020 on a convenience sample of 6 dairy calf rearing facilities across California’s Central Valley. Three of the rearing operations were multi-source (range 8 to approximately 45 farm sources of calves), two were dairies that raised their own calves on farm, and one was a dairy that raised their weaned calves on the source farm after being raised at a multi-source calf rearing facility prior to weaning (number of sources unknown). The study was approved by the UC Davis Institutional Animal Care and Use Committee (protocol # 20114). Informed consent from herd management was obtained verbally prior to commencing study activities.

### Sample collection

The sample size calculation was performed using a 2-sided test, a type 1 error of 0.05, a power of 80%, and an assumed proportion difference of 50% between phenotypic AMR for BRD score positive compared to score negative heifers. A BRD score was assigned as a binary outcome of either positive for clinical signs of BRD or negative for clinical signs of BRD, based on a clinical scoring system validated in weaned heifers [28]. A total of 283 heifers were required, however, to account for a 20% dropout rate due to missing records, at least 340 heifers were required. To achieve similar sampling numbers between two seasons in all 6 farms, 360 animals were enrolled for sampling. Selection criteria included weaned heifers in group pens (≥ 3 months of age) that had been comingled for at least 2 weeks prior to sampling and that were less than 6 months of age based on farm records. Bull or steer calves comingled with heifers were excluded from sampling. Heifers both with and without clinical signs of BRD based on a validated BRD Scoring system for weaned heifers [28] were included. Heifers scoring positive for BRD were enrolled in a convenience sample of all BRD score positive heifers in the pens available for sampling until 15 BRD score positive animals were identified. Heifers classified as BRD score negative, were selected randomly, using a random number generator smartphone application, from the same pens until 15 BRD score negative animals were identified. Samples were collected over two seasonal time points to include warmer (summer /early fall) and cooler (winter/early spring) seasons.

### Sampling procedure

Respiratory bacterial isolates were collected using deep nasopharyngeal swabs (DNPS) (double guarded culture swabs, Reproduction Provisions LLC, Walworth WI, USA) as previously described [29, 30]. Swabs were immediately placed in Amies with charcoal transport media (CultureSwab Plus, BD BBL™, COPAN Italia SpA, Brescia Italy). Enteric bacterial samples were collected from rectal swabs as previously described [31] and swabs were immediately placed in Amies transport media (Transporter® Amies Gel, Hardy Diagnostics, Santa Maria CA USA). All samples were stored in a cooler with ice during sampling, followed by refrigeration at ~4°C for up to 48 h before submission for bacteriological analysis.

### Culture and antimicrobial sensitivity testing

All swabs were submitted to the California Animal Health and Food Safety laboratory in Davis, CA for selective culture and sensitivity testing. Each DNPS in Amies with charcoal transport media was cultured on sheep blood-3% agar (3% SBA) and chocolate agar (CHOC). Rectal swabs in Amies transport media were cultured on MacConkey agar (MAC), mannitol salt agar (MSA) and bile-esculin (BE) plates. Plates were incubated for 48 hours at 35 ± 2˚C in 5–10% CO_2_ (3% SBA, CHOC) or ambient air (MAC, MSA, BE) and examined every 18–24 hours for colonies of interest. Organisms of interest included the respiratory pathogens *Mannheimia haemolytica*, *Pasteurella multocida*, and *Histophilus somni* recovered from DNPS; and *Escherichia coli* and *Enterococcus* spp. recovered from rectal swab cultures to represent Gram negative and Gram positive enteric indicator bacteria, respectively. All colonies of interest were confirmed by biochemical testing and matrix-assisted, laser desorption-ionization time of flight (MALDI-TOF) mass spectrometry.

Antimicrobial susceptibility testing was performed using broth microdilution (Trek Sensititre, Trek Diagnostic Systems, Thermo Fisher Scientific, Waltham, MA) according to Clinical Laboratory Standards Institute (CLSI) guidelines [32] to determine the MIC of the 19 AMD contained on the Sensititre Bovine BOPO7F Vet AST plate (Thermo Scientific, Remel Inc., Lenexa, KS, USA). This panel of 19 AMD was selected to match those monitored by the United States Department of Agriculture, Animal and Plant Health Inspection Service [33, 34]. Where available, interpretive criteria from CLSI-established clinical breakpoints [35] were used to classify an organism as susceptible versus not susceptible (resistant or intermediate). These clinical breakpoints are available only for the respiratory bacterial isolates investigated in this study; no CLSI established breakpoints that are clinically applicable to cattle health exist for enteric isolates obtained from rectal swabs of calves so United States Department of Agriculture (USDA) National Antimicrobial Resistance Monitoring System (NARMS) breakpoints [36] were applied to enteric isolates, where available, for the purpose of discussion of AMR as a potential human zoonosis. Although there are published CLSI breakpoints for ampicillin and sulfadimethoxine for *P*. *multocida*, *M*. *haemolytica*, and *H*. *somni*, the current MIC breakpoints are below the lowest concentration of drug tested in the standard broth microdilution method used, and thus susceptibility could not be meaningfully interpreted based on the MIC provided. The suggested epidemiologic cutoff between wild type and evidence of acquired AMR for *P*. *multocida*, suggested by the European Committee on Antimicrobial Susceptibility Testing, is 1 μg/mL for ampicillin [37]. Isolates were tested against antimicrobials at the following dilutions: ampicillin (0.25–16μg/mL), penicillin (0.12–8 μg/mL), ceftiofur (0.25–8 μg/mL), florfenicol (0.25–8 μg/mL), tylosin (0.5–32 μg/mL), tilmicosin (2–16 μg/mL), tulathromycin (8–64 μg/mL), tildipirosin (2–16 μg/mL), gamithromycin (1–8 μg/mL), tiamulin (0.5–32 μg/mL), clindamycin (0.25–16 μg/mL), danofloxacin (0.12–1 μg/mL), enrofloxacin (0.12–2 μg/mL), trimethoprim-sulfamethoxazole (2/38 μg/mL), sulfadimethoxine (256 μg/mL), tetracycline (0.5–8 μg/mL), gentamicin (1–16 μg/mL), neomycin (8–32 μg/mL), and spectinomycin (8–64 μg/mL). *Escherichia coli* ATTC 25922, *Pseudomonas aeruginosa* ATTC 27853, *Enterococcus faecalis* ATTC 29212, and *Staphylococcus aureus* ATTC 29213 were used as quality control organisms.

Isolates were sub-cultured on SBA (*M*. *haemolytica*, *P*. *multocida*, *E*. *coli*, *Enterococcus* spp.*)* or CHOC (*H*. *somni)* and incubated for 18–24 hours at 35˚C in 5–10% CO_2_. Each isolate was suspended in 0.85% saline to a concentration equivalent to a 0.5 McFarland standard and added to 10ml of cation-adjusted Mueller-Hinton broth (*E*. *coli*, *Enterococcus* spp.), cation-adjusted Mueller-Hinton broth containing lysed horse blood (*M*. *haemolytica*, *P*. *multocida)*, or veterinary fastidious media (*H*. *somni)* to achieve 5 X 10^5^−1 × 10^6^ cfu/mL. Susceptibility plates were incubated for 16–20 hours (*E*. *coli*, *Enterococcus* spp.), 18–24 hours at 35 ± 2˚C in ambient air (*M*. *haemolytica*, *P*. *multocida)*, or 5–10% CO_2_ (*H*. *somni)* and observed for visible growth. The MIC was determined as the lowest concentration of antimicrobial that prevented growth. For AMD in which there was no applicable CLSI breakpoint for the respiratory pathogens tested, and for all enteric isolates tested, the distribution of MIC across the complete range of AMD concentrations tested was described.

Multi-drug resistance was defined as a respiratory isolate and AMD test combination for which there were applicable CLSI breakpoints and the isolate was not susceptible to ≥ 3 AMD classes, as previously described [38]. The 11 AMD with applicable CLSI breakpoints represented 7 classes of AMD. Class-wide susceptibility applied when isolates were susceptible to all AMD tested within a class; lack of class wide susceptibility was applied when an isolate was not susceptible (resistant or intermediate) to one or more of any of the drugs tested within the class.

### Data analyses

Descriptive statistics, including the number of weaned dairy heifers enrolled from each farm, breed, BRD score, and the season in which the samples were collected, were determined. For each AMD and respiratory pathogen combination, the proportion (to provide an estimate of prevalence) of susceptible and not susceptible isolates and their 95% confidence interval (95% CI) were calculated. For all AMD and bacterial isolate combinations for which there was no applicable CLSI breakpoint, the distribution of isolates across the range of MIC tested was described, and enteric isolates were compared with USDA NARMS breakpoints where available. Association between isolation of a resistant respiratory (*P*. *multocida*, *M*. *haemolytica* or *H*. *somni*) pathogen and a high or low MIC of the enteric (*E*. *coli* or *Enterococcus* spp.) isolate from the same calf was determined by construction of 2 × 2 frequency tables followed by calculation of prevalence odds ratio (POR) and 95% CI. In the frequency table, the result of the susceptibility pattern of the respiratory isolate (susceptible or not susceptible to specific AMD) was considered the risk factor, whereas the outcome was the category of MIC (high or low) for the enteric pathogens. Specifically, for each enteric pathogen, for each AMD, MIC were categorized as high or low based on the distribution of isolates in each category. Prevalence odds ratios were considered significant when >1 or <1 with a 95% CI excluding 1, and a corresponding *p*<0.05 was calculated using exact methods. In all comparisons, a statistical software (GraphPad Prism v 8.4.3, GraphPad Software, San Diego, CA) was used.

## Results

### Calves

A total of 360 calves were initially enrolled, however 19 calves were excluded from analysis due to sample label error, record inconsistency, sex not recorded, or older than 6 months of age. The final 341 calves included 239, 98, and 4 Holsteins, Jerseys, and Holstein/Jersey cross-breeds, respectively. A total of 172 and 169 BRD score positive and BRD score negative calves, respectively, were included in the final analysis. In hot weather months (June through October), a mean of 28 calves were enrolled per farm (range 26–30). In cooler weather months (January and February) a mean of 29 calves were enrolled from each farm (range 26–30). The mean total enrollment per farm for both seasons was 57 calves (range 53–60).

### Respiratory pathogens analysis

**Fig 1:**
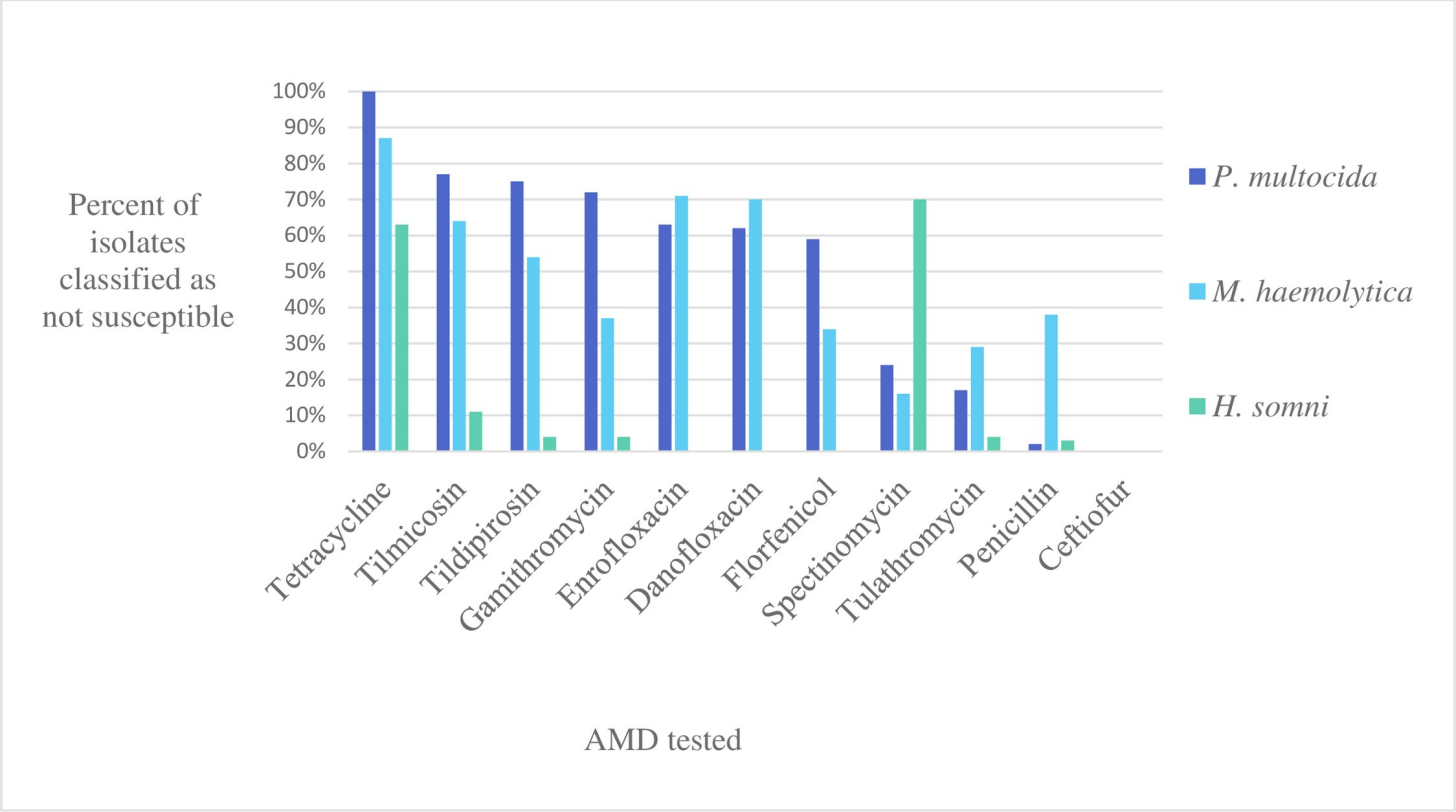
Percent of respiratory isolates classified by CLSI breakpoints as not susceptible (resistant or intermediate) to 11 antimicrobial drugs tested by broth microdilution method. *P*. *multocida* (n = 145), *M*. *haemolytica* (n = 119), *H*. *somni* (n = 97). AMD = antimicrobial drug.

Of the 341 DNPS samples cultured, 263 had one or more respiratory bacterial isolates of interest recovered (145 *P*. *multocida*, 119 *M*. *haemolytica* and 97 *H*. *somni*), for an isolation rate of 77%. Of calves with at least one respiratory isolate of interest, 133 and 130 were BRD score positive and negative, respectively. Lack of AMD susceptibility was common in respiratory isolates (**Fig 1**); 39% of all susceptibility outcomes for respiratory isolates and AMD combinations with CLSI breakpoints were classified as not susceptible. A substantial proportion of *P*. *multocida*, *M*. *haemolytica*, and *H*. *somni* isolates (100, 87 and 61% respectively) lacked susceptibility to tetracycline. In addition, greater than 50% of *P*. *multocida* and *M*. *haemolytica* isolates were classified as not susceptible to tilmicosin, tildipirosin, danofloxacin, enrofloxacin.

#### Pasteurella multocida

All (100%) *P*. *multocida* isolates were classified as susceptible to ceftiofur, and 97.9% (95%CI; 94.1, 99.3) were susceptible to penicillin, 82.8% (75.8, 88.0) to tulathromycin, and 75.9% (95%CI; 68.3, 82.1) to spectinomycin. All (100%) of *P*. *multocida* isolates were classified as not susceptible to tetracycline, and 77.3% (95%CI; 69.8, 83.3) to tilmicosin, 75.2% (95%CI; 67.6, 81.5) to tildipirosin, 71.7% (95%CI; 63.9, 78.4) to gamithromycin, 62.1% (95%CI; 54, 69.6) to danofloxacin, 62.1% (95%CI; 54, 69.6) to enrofloxacin and 59.3% (95%CI; 51.2, 67) to florfenicol (**Fig 1**, **S1 Table**).

#### Mannheimia haemolytica

All (100%) of *M*. *haemolytica* isolates were classified as susceptible to ceftiofur, and 62.2% (95%CI; 53.2, 70.4) to penicillin, 70.6% (95%CI; 61.9, 78.0) to tulathromycin, 84.0% (95%CI; 76.4, 89.5) to spectinomycin, 66.4% (95%CI; 57.5, 74.3) to florfenicol, and 63% (95%CI; 54, 71.2) to gamithromycin. Most, 86.6% (95%CI; 79.3, 91.6) of *M*. *haemolytica* isolates were classified as not susceptible to tetracycline, 63.9% (95%CI; 54.9, 71.9) not susceptible to tilmicosin, 53.8% (95%CI; 44.9, 62.5) not susceptible to tildipirosin, 69.8% (95%CI; 61, 77.3) not susceptible to danofloxacin, and 71.4% (95%CI; 62.7, 78.8) not susceptible to enrofloxacin, (**Fig 1, S1 Table**).

#### Histophilus somni

All (100%) of *H*. *somni* isolates were classified as susceptible to ceftiofur, florfenicol, danofloxacin and enrofloxacin while 96.9% (95%CI; 91.3, 98.9) were classified as susceptible to penicillin, 95.9% (95%CI; 89.9, 98.4) to tulathromycin, 88.7% (95%CI; 89.9, 98.4) to tilmicosin, 95.9% (95%CI; 89.9, 98.4) to gamithromycin, and 95.9% (95%CI; 89.9, 98.4) to tildipirosin. Most, 70.0% (95%CI; 60.4, 78.3), of *H*. *somni* isolates were classified as not susceptible to spectinomycin, and 62.9% (95%CI; 53, 71.4) as not susceptible to tetracycline, (**Fig 1**, **S1 Table**).

The distribution of MIC for all AMD tested, including those for which there was no applicable CLSI breakpoints in the selected respiratory bacteria, are summarized in **Table 1**. The majority of *P*. *multocida*, *M*. *haemolytica*, and *H*. *somni* isolates had MIC at or below the lowest drug concentrations tested for ampicillin (97, 71 and 95% of isolates, respectively) and trimethoprim sulfamethoxazole (92, 97, and 76% of isolates, respectively). The majority of *P*. *multocida* and *M*. *haemolytica* isolates have MIC at or above the highest drug concentrations tested for tylosin (99 and 100% of isolates, respectively), clindamycin (99 and 86% of isolates, respectively), neomycin (98 and 84% of isolates, respectively) and sulfadimethoxine (100 and 88% of isolates, respectively).

**Table 1:** Distribution of MIC of antimicrobial drugs (AMD) as percent of isolates for *E*. *coli*, *Enterococcus* spp., *P*. *multocida*, *M*. *haemolytica*, and *H*. *somni*.

		Percent of isolates at each MIC (μg/mL)																								
Antibiotic	Isolate	≤ 0.12	0.12	≤0.25	0.25	≤0.5	0.5	≤ 1	1	>1	≤2	2	>2	≤4	4	≤8	8	>8	16	>16	32	>32	64	>64	≤ 256	>256
Penicillin	*E*. *coli*	0	0	0	0	0	0	0	0	0	0	0	0	0	0	0	2	98								
	*Enterococcus* spp	15	0	0	4	0	9	0	19	0	0	30	0	0	20	0	4	0								
	*P*. *multocida*	57	0	0	41	0	0	0	0	0	0	0	0	0	0	0	0	2								
	*M*. *haemolytica*	34	0	0	29	0	8	0	1	0	0	0	0	0	0	0	0	29								
	*H*. *somni*	93	0	0	4	0	1	0	0	0	0	0	0	0	0	0	2	0								
Ampicillin	*E*. *coli*	* *		0	0	0	0	0	4	0	0	29	0	0	47	0	3	0	0	17						
	*Enterococcus* spp	* *		24	0	0	24	0	41	0	0	11	0	0	0	0	0	0	0	0						
	*P*. *multocida*	* *		97	0	0	1	0	0	0	0	0	0	0	0	0	1	0	1	0						
	*M*. *haemolytica*	* *		71	0	0	1	0	0	0	0	0	0	0	0	0	1	0	3	25						
	*H*. *somni*	* *		95	0	0	1	0	1	0	0	0	0	0	2	0	0	0	0	1						
Ceftiofur	*E*. *coli*	* *		33	0	0	57	0	1	0	0	0	0	0	0	0	4	4								
	*Enterococcus* spp	* *		2	0	0	7	0	0	0	0	9	0	0	6	0	11	65								
	*P*. *multocida*	* *		99	0	0	0	0	1	0	0	0	0	0	0	0	0	0								
	*M*. *haemolytica*	* *		99	0	0	0	0	0	0	0	1	0	0	0	0	0	0								
	*H*. *somni*	* *		100	0	0	0	0	0	0	0	0	0	0	0	0	0	0								
Florfenicol	*E*. *coli*	* *		0	0	0	0	0	0	0	0	5	0	0	18	0	3	74								
	*Enterococcus* spp	* *		0	0	0	0	0	4	0	0	76	0	0	15	0	0	6								
	*P*. *multocida*	* *		1	0	0	34	0	6	0	0	0	0	0	3	0	37	19								
	*M*. *haemolytica*	* *		0	0	0	8	0	27	0	0	32	0	0	0	0	2	32								
	*H*. *somni*	* *		63	0	0	19	0	19	0	0	0	0	0	0	0	0	0								
Tylosin	*E*. *coli*	* *				0	0	0	0	0	0	0	0	0	0	0	0	0	0	0	0	100				
	*Enterococcus* spp	* *				2	0	0	0	0	0	63	0	0	7	0	2	0	0	0	0	26				
	*P*. *multocida*	* *				0	0	0	0	0	0	0	0	0	0	0	0	0	0	0	9	91				
	*M*. *haemolytica*	* *				0	0	0	0	0	0	0	0	0	0	0	0	0	0	0	7	93				
	*H*. *somni*	* *				0	0	0	0	0	0	3	0	0	19	0	52	0	19	0	4	4				
Tilmicosin	*E*. *coli*	* *				0	0	0	0	0	0	0	0	0	0	0	0	0	2	98						
	*Enterococcus* spp	* *				0	0	0	0	0	0	0	0	0	4	0	50	0	20	26						
	*P*. *multocida*	* *				0	0	0	0	0	0	0	0	0	8	0	15	0	3	74						
	*M*. *haemolytica*	* *				0	0	0	0	0	0	0	0	0	24	0	13	0	14	50						
	*H*. *somni*	* *				0	0	0	0	0	0	18	0	0	31	0	40	0	7	4						
Tulathromycin	*E*. *coli*	* *														79	0	0	20	0	1	0	0	0		
	*Enterococcus* spp	* *														70	0	0	4	0	0		0	26		
	*P*. *multocida*	* *														54	0	0	29	0	8	0	4	6		
	*M*. *haemolytica*	* *														45	0	0	26	0	9	0	3	17		
	*H*. *somni*	* *														85	0	0	11	0	0	0	0	4		
Tildipirosin	*E*. *coli*	* *						1	0	0	0	11	0	0	70	0	17	0	1	0						
	*Enterococcus* spp	* *						0	0	0	0	0	0	0	0	0	28	0	43	30						
	*P*. *multocida*	* *						22	0	0	0	3	0	0	0	0	0	0	0	75						
	*M*. *haemolytica*	* *						31	0	0	0	12	0	0	3	0	3	0	21	30						
	*H*. *somni*	* *						25	0	0	0	34	0	0	26	0	11	0	0	4						
Gamithromycin	*E*. *coli*	* *						0	0	0	0	3	0	0	23	0	61	14								
	*Enterococcus* spp	* *						63	0	0	0	7	0	0	4	0	4	22								
	*P*. *multocida*	* *						22	0	0	0	2	0	0	4	0	45	27								
	*M*. *haemolytica*	* *						32	0	0	0	20	0	0	11	0	12	25								
	*H*. *somni*	* *						94	0	0	0	0	0	0	2	0	0	4								
Tiamulin	*E*. *coli*	* *				0	0	0	0	0	0	0	0	0	0	0	0	0	2	0	14	84				
	*Enterococcus* spp	* *				15	0	0	17	0	0	7	0	0	0	0	2	0	2	0	2	56				
	*P*. *multocida*	* *				0	0	0	0	0	0	0	0	0	0	0	0	0	19		76	5				
	*M*. *haemolytica*	* *				0	0	0	0	0	0	0	0	0	0	0	34	0	29	0	29	8				
	*H*. *somni*	* *				7	0	0	44	0	0	45	0	0	3	0	0	0	0	0	0	0				
Clindamycin	*E*. *coli*	* *		0	0	0	0	0	0	0	0	0	0	0	0	0	0	0	0	100						
	*Enterococcus* spp	* *		28	0	0	4	0	0	0	0	7	0	0	4	0	9	0	26	22						
	*P*. *multocida*	* *		0	0	0	0	0	0	0	0	0	0	0	0	0	1	0	0	99						
	*M*. *Haemolytica*	* *		0	0	0	0	0	0	0	0	0	0	0	0	0	14	0	59	27						
	*H*. *somni*	* *		7	0	0	21	0	49	0	0	18	0	0	0	0	1	0	0	4						
Danofloxacin	*E*. *coli*	78	0	0	4	0	5	0	3	10																
	*Enterococcus* spp	0	0	0	11	0	48	0	24	17																
	*P*. *multocida*	37	0	0	1	0	0	0	8	54																
	*M*. *haemolytica*	26	0	0	4	0	44	0	0	26																
	*H*. *somni*	100	0	0	0	0	0	0	0	0																
Enrofloxacin	*E*. *coli*	78	0	0	4	0	4	0	6	0	0	1	7													
	*Enterococcus* spp	0	0	0	13	0	59	0	17	0	0	6	6													
	*P*. *multocida*	38	0	0	0	0	5	0	27	0	0	23	8													
	*M*. *haemolytica*	26	0	0	3	0	44	0	11	0	0	5	12													
	*H*. *somni*	100	0	0	0	0	0	0	0	0	0	0	0													
Tetracycline	*E*. *coli*	* *				0	0	0	14	0	0	4	0	0	0	0	0	82								
	*Enterococcus* spp	* *				28	0	0	7	0	0	2	0	0	0	0	0	63								
	*P*. *multocida*	* *				0	0	0	0	0	0	0	0	0	1	0	10	90								
	*M*. *haemolytica*	* *				13	0	0	0	0	0	0	0	0	28	0	20	39								
	*H*. *somni*	* *				7	0	0	10	0	0	20	0	0	32	0	13	18								
Gentamicin	*E*. *coli*	* *						94	0	0	0	4	0	0	1	0	2	0	0	0						
	*Enterococcus* spp	* *						0	0	0	0	2	0	0	33	0	48	0	17	0						
	*P*. *multocida*	* *						1	0	0	0	17	0	0	78	0	3	0	1	0						
	*M*. *haemolytica*	* *						0	0	0	0	44	0	0	50	0	0		5	1						
	*H*. *somni*	* *						15	0	0	0	7	0	0	16	0	36	0	20	5						
Neomycin	*E*. *coli*	* *												79	0	0	0	0	1	0	4	16				
	*Enterococcus* spp	* *												4	0	0	13	0	30	0	17	37				
	*P*. *multocida*	* *												0	0	0	1	0	1	0	6	92				
	*M*. *haemolytica*	* *												6	0	0	9	0	1	0	3	81				
	*H*. *somni*	* *												3	0	0	2	0	12	0	34	48				
Spectinomycin	*E*. *coli*	* *														5	0	0	61	0	6	0	1	26		
	*Enterococcus* spp	* *														0	0	0	4	0	9	0	81	6		
	*P*. *multocida*	* *														1	0	0	19	0	55	0	0	24		
	*M*. *haemolytica*	* *														1	0	0	14	0	69	0	0	16		
	*H*. *somni*	* *														0	8	0	18	0	4	0	4	66		
Trimethoprim sulfamethoxazole	*E*. *coli*	* *									75		25													
	*Enterococcus* spp	* *									83		17													
	*P*. *multocida*	* *									92		8													
	*M*. *haemolytica*	* *									97		3													
	*H*. *somni*	* *									76		24													
Sulpha-dimethoxine	*E*. *coli*	* *																							22	78
	*Enterococcus* spp	* *																							0	100
	*P*. *multocida*	* *																							0	100
	*M*. *haemolytica*	* *																							12	88
	*H*. *somni*	* *																							59	41

*E*. *coli* (n = 341), *Enterococcus* spp. (n = 54), *P*. *multocida* (n = 145), *M*. *haemolytica* (n = 119), and *H*. *somni* (n = 97). Gray shading represents limits of the ranges of AMD concentrations tested for each drug. Each cell is graded by color intensity; darkest blue represents the greatest number of isolates. Breakpoints for susceptibility are shown as thick lines where applicable; CLSI breakpoints are used for respiratory isolates and USDA NARMS breakpoints are used for enteric isolates.

### Enteric pathogens

All rectal swabs yielded *E*. *coli* isolates (n = 341), whereas 54 swabs yielded *Enterococcus* spp. (n = 54). Most *E*. *coli* isolates’ MIC were at or above the highest concentration tested for: penicillin (100% of isolates), florfenicol (77% of isolates), tylosin (100% of isolates), tilmicosin (100% of isolates), gamithromycin (75% of isolates), tiamulin (98% of isolates), clindamycin (100% of isolates), tetracycline (82% of isolates), and sulfadimethoxine (78% of isolates). In contrast, most *E*. *coli* isolates’ MIC were at or below the lowest end of the concentrations tested for: tulathromycin (79% of isolates), enrofloxacin (78% of isolates), danofloxacin (78% of isolates), gentamicin (94% of isolates), neomycin (79% of isolates) and trimethoprim sulfamethoxazole (75% of isolates) (**Table 1**). Most *Enterococcus* spp. isolates’ MIC were at or above the highest concentration of drug tested for: ceftiofur (76% of isolates), tildipirosin (73%), tiamulin (58% of isolates), tetracycline (63% of isolates), neomycin (54% of isolates), spectinomycin (87% of isolates) and sulfadimethoxine (100% of isolates). In contrast, most *Enterococcus* spp. isolates’ MIC were at or below the lowest concentration tested for: tulathromycin (70% of isolates), gamithromycin (63% of isolates), and trimethoprim sulfamethoxazole (83% of isolates) (**Table 1**). Using USDA NARMS breakpoints, all *Enterococcus* spp. isolates in this study were classified as susceptible to penicillin however only 74% are classified as susceptible to tylosin, and only 37% susceptible to tetracycline. Using the USDA NARMS breakpoints, 98% of *E*. *coli* isolates were classified as susceptible to gentamicin, however only 83% were classified as susceptible to ampicillin, 75% as susceptible to trimethoprim sulfamethoxazole, 22% as susceptible to sulfadimethoxine, and 18% as susceptible to tetracycline.

### Respiratory bacterial isolate breakpoint interpretation relationship to enteric bacterial isolate MIC

The distribution of MIC for all bacterial isolates tested are displayed in **Table 1**. For calves where both *M*. *haemolytica* and *E*.*coli* were isolated, the POR of a higher MIC (>4 μg/mL) for *E*.*coli* was 2.64 (95%CI; 1.07, 6.62; *P* = 0.0385) times for *M*. *haemolytica* isolates that were classified as not susceptible to gamithromycin. For calves where both *P*. *multocida* and *E*.*coli* were isolated, the POR of a higher MIC (>8 μg/mL) for *E*.*coli* was 2.39 (95% CI, 1.06, 5.14; *P* = 0.0331) times for *P*. *multocida* isolates that were classified as not susceptible to tulathromycin. There was no association between isolation of a respiratory bacteria classified as not susceptible by CLSI breakpoint interpretation and the classification of enteric bacterial MIC as high or low for any other combinations of respiratory isolate, enteric isolate, or AMD tested for those AMD with an applicable CLSI breakpoint in the respiratory isolates.

### Multi-drug resistance profiles

**Fig 2:**
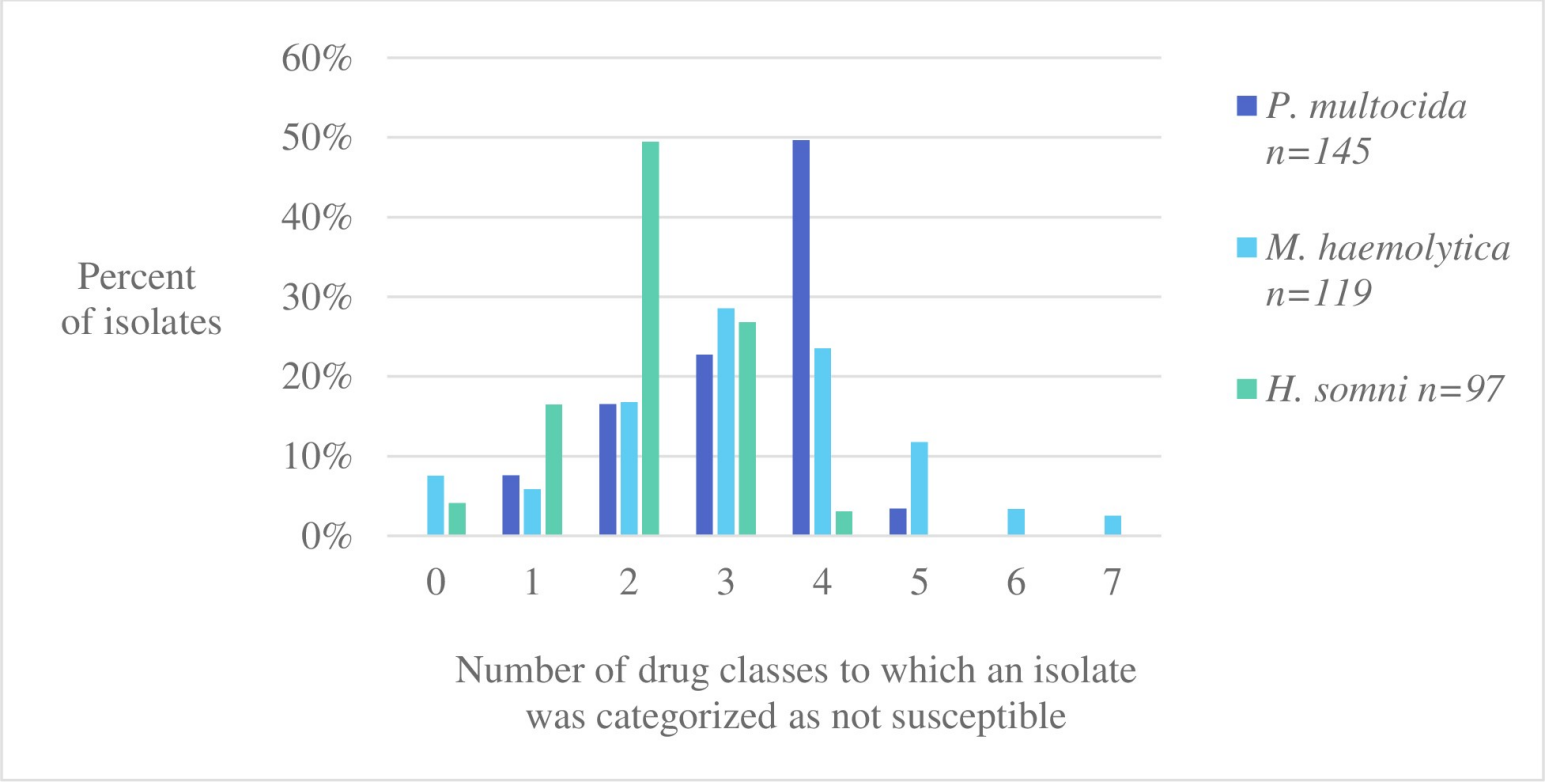
Number of antimicrobial drug classes to which each respiratory isolate (*P*. *multocida*, *M*. *haemolytica* and *H*. *somni*) was classified as not susceptible (resistant or intermediate). The distribution of isolates by number of classes to which they were categorized as not susceptible is shown as percent of total isolates per species. Seven drug classes were represented among antimicrobial drugs with applicable breakpoints. *P*. *multocida* (n = 145), *M*. *haemolytica* (n = 119), *H*. *somni* (n = 97).

Multidrug resistance was present in 76% (110/145), 70% (83/119), and 30% (29/97) of *P*. *multocida*, *M*. *haemolytica* and *H*. *somni* isolates respectively. **Fig 2** depicts the distribution of respiratory isolates by the number of antimicrobial drug classes to which they were classified as not susceptible, between 1 and 7 classes of AMD tested.

**Fig 3:**
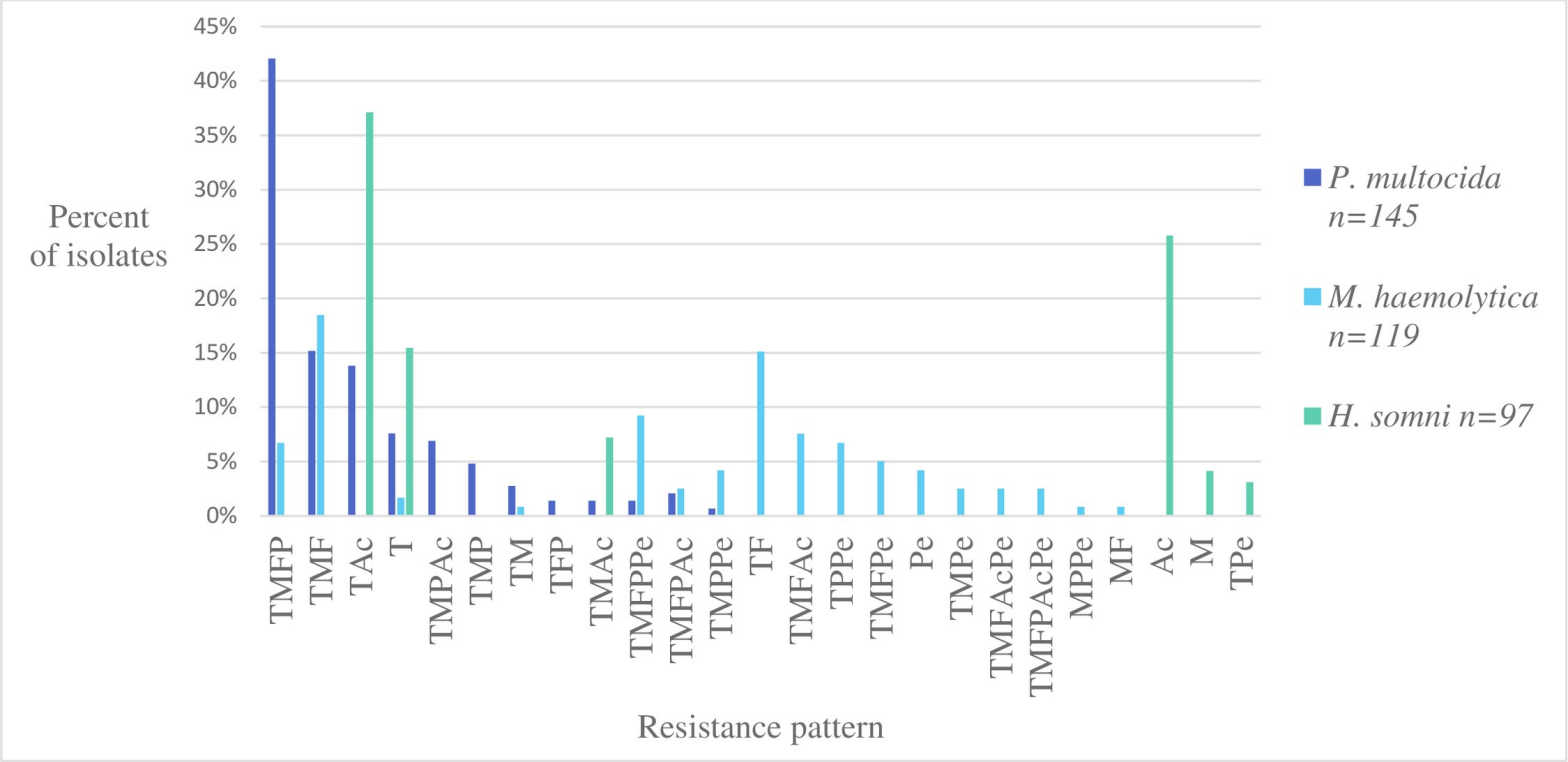
Percent of *P*. *multocida*, *M*. *haemolytica* and *H*. *somni* isolates demonstrating each of 26 total resistance patterns. Each pattern is defined by lack of susceptibility to one or more drug classes using the following abbreviations: T = tetracycline, M = macrolides, F = fluoroquinolones, P = phenicol, Ac = aminocyclitol, Pe = penicillin. *P*. *multocida* (n = 145), *M*. *haemolytica* (n = 119), *H*. *somni* (n = 97).

Twelve, 17 and 6 AMR patterns were demonstrated by *P*. *multocida*, *M*. *haemolytica* and *H*. *somni* isolates, respectively (**Fig 3)**. Patterns of MDR displayed by both *P*. *multocida* and *M*. *haemolytica* included tetracycline, macrolides, and fluoroquinolone with or without phenicol resistance. The most prevalent MDR phenotype was resistance to tetracycline, macrolides, fluoroquinolones, and phenicol in *P*. *multocida* isolates, followed by tetracycline, macrolide and fluoroquinolone resistance in *M*. *haemolytica* isolates, and tetracycline, aminocyclitol resistance in *H*. *somni* isolates. Tetracycline resistance was the most common drug resistance present in patterns of MDR (**Fig 3**).

## Discussion

The most prevalent AMR phenotype, using applicable CLSI breakpoints, in the respiratory isolates studied was against tetracycline. This finding is consistent with previous reports of susceptibility by respiratory bacterial isolates in other cattle populations [39]. Resistance to tetracycline in addition to other AMD was common in our study; out of 25 different patterns of AMR or MDR, 20 patterns include tetracycline resistance. In addition to tetracycline, *P*. *multocida* and *M*. *haemolytica* were also frequently (>50% of isolates) classified as not susceptible to the macrolides (tildipirosin and tilmicosin), and fluoroquinolones (danofloxacin and enrofloxacin). *H*. *somni* isolates were less frequently classified as not susceptible, and lack of susceptibility was most frequent for tetracycline and spectinomycin.

The similarity between AMR patterns in *P*. *multocida* and *M*. *haemolytica* might be a reflection of the presence of both bacteria in the nasopharynx of calves, and exposure to the same pressures that influence the development of AMR (16). Furthermore, the bacteria may transfer mechanisms of resistance between bacterial species via mobile genetic elements [24]. In contrast, *H*. *somni* is less commonly isolated from the nasopharynx of calves [40], and therefore may be less frequently exposed to the same pressures that influence development of AMR, or may have less contact and thus be less likely to share resistance mechanisms compared to *P*. *multocida* and *M*. *haemolytica*.

Similar to respiratory isolates, enteric bacterial MIC were consistently high for tetracycline; greater than 50% of both *E*. *coli* and *Enterococcus* spp. isolates had MIC at the highest concentration of tetracycline tested. If the USDA NARMS breakpoint of 4μg/mL is applied, 82 and 63% of *E*. *coli* and *Enterococcus* spp. isolates, respectively, would be considered resistant. The *E*. *coli* isolates in the study population also demonstrated AMR, according to USDA NARMS breakpoints, to gentamicin, ampicillin, trimethoprim sulfamethoxazole and sulfadimethoxine in 2, 17, 25 and 78% of samples, respectively. The *Enterococcus* spp. isolates in the study population also demonstrated AMR, according to USDA NARMS breakpoints, to tylosin in 26% of samples. These finding raise concern for AMR in these enteric marker bacteria, particularly for tetracycline in both types of isolates, and sulfonamide AMD in *E*. *coli* and tylosin in *Enterococcus* spp. Although these NARMS breakpoints are not clinically applicable to disease in the calves from which they were isolated, these findings may have implications for human health and zoonotic transmission of bovine enteric bacteria to humans. Gentamicin and trimethoprim sulfamethoxazole are not labeled for use in cattle and there is a voluntary ban on the use of aminoglycosides (gentamicin) in cattle in the United States due to prolonged tissue drug residues [41].

The relationship between AMR in respiratory isolates to higher MIC of enteric marker bacteria appeared to be limited. The only significant relationships identified in this study were between *M*. *haemolytica* resistance to gamithromycin and a higher MIC for gamithromycin in *E*. *coli*; and *P*. *multocida* resistance to tulathromycin and a higher MIC for tulathromycin in *E*. *coli*. This association suggests that the susceptibility phenotypes to gamithromycin and tulathromycin of both respiratory and enteric bacteria may be similarly affected by selection pressures within the host, or may share AMR mechanisms for these two macrolide drugs. The lack of association between respiratory bacterial AMR and a higher enteric bacterial MIC for the other AMD tested suggests that their relative susceptibility is not directly associated in matched bacterial samples. This is surprising because the enteric bacteria exist in the same host, are exposed to the same environmental and host factors, and have previously been demonstrated to share AMR mechanisms *in vitro* [24]. This difference may be due to different responses to selective pressures between the two populations of bacteria (upper respiratory *vs* enteric). Isolates may lose acquired AMR over time after treatment ceases, and differences in drug distribution between tissues and body sites likely effect the time it takes for drug concentrations to decrease after treatment. Previous investigations in beef and dairy cattle populations have identified changes in both respiratory and enteric bacterial populations and their associated AMR elements associated with AMD treatment; these changes are related to type or route of AMD administration, vary by production system being investigated, and are not uniform between respiratory and enteric bacterial populations [42–44]. Additionally, herd dynamics and the spread of resistant bacteria or mobile genetic elements between animals should be considered. Previous investigations in feedlot cattle have suggested AMR spreads with comingling [45].

Our study findings are clinically significant because they demonstrate the widespread existence of AMR in a significant, but relatively understudied, population of animals. There are many host, pathogen and environmental factors that may affect treatment failure, and AMR is one concerning factor. The problem of AMR may be compounded when more AMD are used in an attempt to overcome AMR or treatment failure. Of particular concern is the identification of AMR in over half the *P*. *multocida* isolates tested to 7 AMD commonly used for treatment of BRD in weaned heifers (florfenicol, gamithromycin, tildipirosin, tilmicosin, danofloxacin, enrofloxacin and tetracycline). Although no consensus for a maximum threshold for an expected proportion of resistant isolates in a given population has been established in the published literature, these findings raise significant concern for AMR in respiratory bacterial isolates in weaned growing cattle against AMD used commonly to treat or control BRD. The impact of AMR on treatment outcomes in this population is unknown, but warrants further investigation. The treatment or retreatment of calves in comingled weaned populations appears highly likely to be complicated by respiratory bacteria resistant to most AMD commonly used for this purpose. Continued use of these medically important AMD is expected to maintain AMR in this population, however it is unknown if decreasing or ceasing the use of these AMD in this population will be sufficient to significantly reduce AMR in this population. Almost all respiratory isolates were classified as resistant to tetracycline, so it is not recommended to use tetracycline to treat or control BRD in weaned dairy heifers. The recommendation against using AMD known to have low susceptibility is made not only based on presumed lack of efficacy in animals necessitating treatment, but also to prevent increasing selective pressure for MDR, since the tetracycline resistant organism may also harbor genetic elements that confer AMR to other AMD in addition to tetracycline. In this study, resistance to tetracycline in combination with other AMD was common in weaned dairy heifers. The AMD to which *P*. *multocida* was less frequently classified as resistant include the β-lactams penicillin and ceftiofur, as well as tulathromycin and spectinomycin. It should be noted, that these AMD may not be suitable alternatives for treating BRD in heifers for several reasons. The β-lactam class of AMD may frequently be effective against family Pasteurellaceae tested *in-vitro* in this investigation, but these AMD are inherently ineffective for *Mycoplasma* organisms that are also commonly present in the BRD complex of calves [39]; *Mycoplasma* spp were not investigated in this study. Spectinomycin is no longer marketed for use in cattle in the United States. Although lower percentages of AMR were reported for respiratory pathogens (17.2, 29.4, and 4.1% for *P*. *multocida*, *M*. *haemolytica*, and *H*. *somni* isolates, respectively) against tulathromycin in comparison to other macrolide drugs, the reported percentages of AMR may still be a concern due to its wide spread usage for treatment of BRD. The overwhelming prevalence of AMR in respiratory isolates to many AMD commonly used for BRD treatment or control underscores the paramount importance of BRD prevention in calves, particularly prior to entering mixed pens.

### Limitations

The current study was conducted using a convenience sample of calf raising facilities in California’s Central Valley. This cross sectional study was not intended to estimate the duration of AMR, so further longitudinal studies are warranted to investigate the duration of the AMR phenotypes, and if any are maintained until the animal enters lactation. A limitation of this investigation is the *in-vitro* nature of susceptibility testing; *in-vivo* response to treatment might differ due to a variety of host, pathogen and environmental factors. Furthermore, the breakpoints for defining susceptibility were not available for all bacteria/drug combinations tested or compared. When breakpoints for susceptibility interpretation are not available, knowledge of the MIC distribution of a particular organism in a population may provide information about the susceptibility of an individual isolate in question relative to the population. The current study determined the phenotypic AMR pattern among the common respiratory bacterial pathogens in weaned dairy heifers against the commonly used AMD. It was beyond the scope of this study, and further studies are required, to determine the underlying causes of AMR in weaned dairy heifers and the effect of AMD treatment history and management protocols on development of AMR. The data reported herein provide a baseline for understanding AMR among bacterial organisms associated with BRD in California’s heifer rearing operations. Ongoing investigations in this animal population will study the relationship of animal, farm, and environmental factors to the AMR patterns reported herein.

### Conclusions

This cross-sectional study reports the proportion of resistance (lack of susceptibility) in the respiratory bacterial isolates *P*. *multocida*, *M*. *haemolytica*, and *H*. *somni* in weaned dairy heifers in California. Additionally, this study reports the MIC distribution for enteric indicator bacteria *E*. *coli* and *Enterococcus* to the same panel of AMD. The proportions of AMR observed in this study suggest widespread lack of susceptibility (>50% of isolates tested) of *P*. *multocida* and *M*. *haemolytica* to many AMD commonly used for treatment or control of BRD including: tildipirosin, tilmicosin, danofloxacin, enrofloxacin and tetracycline. Most (>50%) *P*. *multocida* isolates were also classified as not susceptible to florfenicol and gamithromycin. Although lack of susceptibility was less frequently identified in *H*. *somni* isolates; most (>50%) of the *H*. *somni* isolates were classified as not susceptible to tetracycline and spectinomycin. Multidrug resistance was common in respiratory isolates, making up 76% (110/145), 70% (83/119), and 30% (29/97) of *P*. *multocida*, *M*. *haemolytica* and *H*. *somni* isolates respectively. The POR for the association between AMR in respiratory isolates and the MIC of enteric indicator bacteria in the same animal demonstrated that for *E*.*coli* the POR of a higher MIC was greater when *M*. *haemolytica* isolates were classified as not susceptible to gamithromycin, and when *P*. *multocida* isolates were classified as not susceptible to tulathromycin. Outside of these associations, respiratory classification of resistance appears to have no significant association with enteric MIC at the same time point. The frequent classification of AMR and MDR in respiratory isolates from a cross-sectional sample of weaned heifers suggests a potentially serious problem of AMR in respiratory pathogens of this population of animals and warrants further investigation and improved BRD prevention in this population.

## Supporting information

S1 File MIC data set for *P*. *multocida*, *M*. *haemolytica*, *H*. *somni*, *E*. *coli* and *Enterococcus* spp. isolates obtained from n = 341 heifers and tested for AMD susceptibility via broth microdilution that were included in study analyses. (XLSX)

S1 Table Proportion (and 95% CI) of *P*. *multocida*, *M*. *haemolytica* and *H*. *somni* isolates classified by applicable CLSI breakpoints as susceptible or not susceptible (resistant or intermediate) to 11 antimicrobial drugs. Green: >50% of isolates classified as susceptible, Red: > 50% of isolates classified as not susceptible.
(DOCX)

S2 Table Breakpoints for susceptibility interpretation of *P*. *multocida*, *M*. *haemolytica* and *H*. *somni* used in study analyses. Source: CLSI VET01 5^th^ ed (Ref 32 in manuscript).
(DOCX)

S3 Table Breakpoints for susceptibility interpretation of *E*. *coli* and *Enterococcus* spp. used in study analyses. Source for *E*. *coli* susceptibility interpretation: https://www.ars.usda.gov/ARSUserFiles/60400520/NARMS/NARMS2009/II.%20Sampling%20and%20Testing%20Methods.pdf. Source for *Enterococcuss* spp. interpretation: https://www.ars.usda.gov/ARSUserFiles/60400520/NARMS/ABXEntero.
(DOCX)
