# Supplementary material for: In-vitro antibiotic resistance phenotypes of respiratory and enteric bacterial isolates from weaned dairy heifers in California
Source: PLoS One. 2021 Nov 24;16(11):e0260292. doi: 10.1371/journal.pone.0260292 (PMC8612539; doi:10.1371/journal.pone.0260292)
Supplement: S1 Table — Green: >50% of isolates classified as susceptible, Red: > 50% of isolates classified as not susceptible. (DOCX) [file pone.0260292.s002.docx]

**S1 Table.** **Proportion (and 95% CI) of *P. multocida, M. haemolytica* and *H. somni* isolates classified by applicable CLSI breakpoints as susceptible or not susceptible (resistant or intermediate) to 11 antimicrobial drugs.**

Green: >50% of isolates classified as susceptible, Red: > 50% of isolates classified as not susceptible.

| Drug | *P. multocida* (n =145) | | *M. haemolytica* (n = 119) | | *H. somni* (n=97) | |
| --- | --- | --- | --- | --- | --- | --- |
|  | Susceptible | Not susceptible | Susceptible | Not susceptible | Susceptible | Not susceptible |
| Penicillin | 97.9  (94.1 – 99.3) | 2.1  (0.7 – 5.9) | 62.2  (53.2–70.4) | 37.8  (29.6 –46.8) | 96.9  (91.3 – 98.9) | 3.1  (1.1 – 8.7) |
| Ceftiofur | 100 | 0 | 100 | 0 | 100 | 0 |
| Florfenicol | 40.7  (33 – 48.8) | 59.3  (51.2 – 67) | 66.4  (57.5 – 74.3) | 33.6  (25.8 –42.5) | 100 | 0 |
| Gamithromycin | 28.3  (21.6 -36.1) | 71.7  (63.9 –78.4) | 63  (54 – 71.2) | 37  (28.8 – 46) | 95.9  (89.9 – 98.4) | 4.1  (1.6 – 10.1) |
| Tildipirosin | 24.8  (18.5 – 32.5) | 75.2  (67.6 –81.5) | 46.2  (37.5 – 55.2) | 53.8  (44.9 –62.5) | 95.9  (89.9 – 98.4) | 4.1  (1.6 – 10.1) |
| Tilmicosin | 22.7  (16.7 – 30.2) | 77.3  (69.8 –83.3) | 36.1  (28.1 – 45.1) | 63.9  (54.9 –71.9) | 88.7  (80.8 – 93.6) | 11.3  (6.5 – 19.2) |
| Tulathromycin | 82.8  (75.8 – 88.0) | 17.2  (12 – 24.2) | 70.6  (61.9 – 78.0) | 29.4  (22 – 38.1) | 95.9  (89.9 – 98.4) | 4.1  (1.6 – 10.1) |
| Danofloxacin | 37.9  (30.4 – 46.0) | 62.1  (54 – 69.6) | 30.2  (22.7 – 39.0) | 69.8  (61 – 77.3) | 100 | 0 |
| Enrofloxacin | 37.9  (30.4 – 46.0) | 62.1  (54 – 69.6) | 28.6  (21.2– 37.3) | 71.4  (62.7 –78.8) | 100 | 0 |
| Tetracycline | 0 | 100 | 13.4  (8.5 – 20.7) | 86.6  (79.3 –91.6) | 37.1  (28.2 – 47.1) | 62.9  (53 – 71.4) |
| Spectinomycin | 75.9  (68.3 – 82.1) | 24.1  (17.9 –31.7) | 84.0  (76.4 – 89.5) | 16.0  (10.5 –23.6) | 30.0  (21.7 – 39.6) | 70.0  (60.4 -78.3) |
