# Supplementary material for: In-vitro antibiotic resistance phenotypes of respiratory and enteric bacterial isolates from weaned dairy heifers in California
Source: PLoS One. 2021 Nov 24;16(11):e0260292. doi: 10.1371/journal.pone.0260292 (PMC8612539; doi:10.1371/journal.pone.0260292)
Supplement: S2 Table — Source: CLSI VET01 5th ed (Ref 32 in manuscript). (DOCX) [file pone.0260292.s003.docx]

**S2. Table. Breakpoints for susceptibility interpretation of *P. multocida, M. haemolytica* and *H. somni* used in study analyses.**

Source: CLSI VET01 5^th^ ed (Ref 32 in manuscript)

|  | ***P. multocida*** | | | ***M. haemolytica*** | | | ***H. somni*** | | |
| --- | --- | --- | --- | --- | --- | --- | --- | --- | --- |
| **Antimicrobial** | **Sensitive** | **Intermediate** | **Resistant** | **Sensitive** | **Intermediate** | **Resistant** | **Sensitive** | **Intermediate** | **Resistant** |
| **Penicillins** |  |  |  |  |  |  |  |  |  |
| Penicillin | ≤0.25 | 0.5 | ≥1 | ≤0.25 | 0.5 | ≥1 | ≤0.25 | 0.5 | ≥1 |
| **Cephalosporins** |  |  |  |  |  |  |  |  |  |
| Ceftiofur | ≤2 | 4 | ≥8 | ≤2 | 4 | ≥8 | ≤2 | 4 | ≥8 |
| **Fluoroquinolones** |  |  |  |  |  |  |  |  |  |
| Danofloxacin | ≤0.25 | 0.5 | ≥1 | ≤0.25 | 0.5 | ≥1 | *extrapolated from M. haemolytica* | | |
| Enrofloxacin | ≤0.25 | 0.5-1.0 | ≥2 | ≤0.25 | 0.5-1.0 | ≥2 | ≤0.25 | 0.5-1.0 | ≥2 |
| **Macrolides** |  |  |  |  |  |  |  |  |  |
| Tilmicosin | *extrapolated from M. haemolytica* | | | ≤8 | 16 | ≥32 | *extrapolated from M. haemolytica* | | |
| Tildipirosin | ≤8 | 16 | ≥32 | ≤4 | 8 | ≥16 | ≤8 | 16 | ≥32 |
| Tulathromycin | ≤16 | 32 | ≥64 | ≤16 | 32 | ≥64 | ≤16 | 32 | ≥64 |
| Gamithromycin | ≤4 | 8 | ≥16 | ≤4 | 8 | ≥16 | ≤4 | 8 | ≥16 |
| **Phenicols** |  |  |  |  |  |  |  |  |  |
| Florfenicol | ≤2 | 4 | ≥8 | ≤2 | 4 | ≥8 | ≤2 | 4 | ≥8 |
| **Tetracyclines** |  |  |  |  |  |  |  |  |  |
| Tetracycline | ≤2 | 4 | ≥8 | ≤2 | 4 | ≥8 | ≤2 | 4 | ≥8 |
| **Aminoglycosides** |  |  |  |  |  |  |  |  |  |
| Spectinomycin | ≤32 | 64 | ≥128 | ≤32 | 64 | ≥128 | ≤32 | 64 | ≥128 |
