# Supplementary material for: In-vitro antibiotic resistance phenotypes of respiratory and enteric bacterial isolates from weaned dairy heifers in California
Source: PLoS One. 2021 Nov 24;16(11):e0260292. doi: 10.1371/journal.pone.0260292 (PMC8612539; doi:10.1371/journal.pone.0260292)
Supplement: S3 Table — Source for E. coli susceptibility interpretation: https://www.ars.usda.gov/ARSUserFiles/60400520/NARMS/NARMS2009/II.%20Sampling%20and%20Testing%20Methods.pdf. Source for Enterococcuss spp. interpretation: https://www.ars.usda.gov/ARSUserFiles/60400520/NARMS/ABXEntero. (DOCX) [file pone.0260292.s004.docx]

**S3. Table. Breakpoints for susceptibility interpretation of *E. coli*  and *Enterococcus* spp. used in study analyses.**

Source for *E. coli*  susceptibility interpretation: <https://www.ars.usda.gov/ARSUserFiles/60400520/NARMS/NARMS2009/II.%20Sampling%20and%20Testing%20Methods.pdf>.

Source for *Enterococcuss* spp. interpretation: https://www.ars.usda.gov/ARSUserFiles/60400520/NARMS/ABXEntero.pdf

|  | ***E. coli*** | | | ***Enterococcus* spp.** | | |
| --- | --- | --- | --- | --- | --- | --- |
| **Antimicrobial drug** | **Sensitive** | **Intermediate** | **Resistant** | **Sensitive** | **intermediate** | **resistant** |
| **Penicillins** |  |  |  |  |  |  |
| Penicillin |  |  |  | <8 | NA | >16 |
| Ampicillin | <8 | 16 | >32 |  |  |  |
| **Macrolides** |  |  |  |  |  |  |
| Tylosin |  |  |  | <8 | 16 | >32 |
| **Tetracyclines** |  |  |  |  |  |  |
| Tetracycline | <4 | 8 | >16 | <4 | 8 | >16 |
| **Aminoglycosides** |  |  |  |  |  |  |
| Gentamicin | <4 | 8 | >16 |  |  |  |
| **Sulfonamides** |  |  |  |  |  |  |
| Trimethoprim/sulfamethoxazole | <2/38 | NA | >4/76 |  |  |  |
